# The Medical Society of the County of Erie

**Published:** 1909-02

**Authors:** Franklin C. Gram

**Affiliations:** Secretary


### SOCIETY PROCEEDINGS.

---

### The Medical Society of the County of Erie.

### Eighty-Seventh Annual Meeting.

REPORTED BY FRANKLIN C. GRAM, M. D. Secretary.

(Carbon copy received January 5, 1909.)

THE eighty-seventh annual meeting of the Medical Society of the County of Erie was held December 21, 1908, in the rooms of the Buffalo Society of Natural Sciences, Public Library Building. The first session was held at 4 o'clock and the second at 8.30 o'clock, p. m., with the president, Dr. Edward Clark, in the chair.

The minutes of the October meeting, also the minutes of the Council meeting held December 7, 1908, were read and approved.

The next order of business being the election of officers for the year 1909, the president appointed Drs. Callanan and Hendee as tellers. The secretary was directed to read the nominations as made at the June meeting.

On separate motions, the secretary was directed to cast the ballot of the society for each of the following officers:

President, Dr. Charles A. Wall; first vice-president, Grover W. Wende; second vice-president, Bernard Cohen.

The president was directed, on motion, to cast the ballot of the Society for Franklin C. Gram as secretary.

Secretary was then directed to cast the ballot of the society for Albert T. Lytle as treasurer.

The president declared each of the foregoing officers to be duly elected for the year 1909.

When the vote was about to be taken for censors, Dr. Henry R. Hopkins arose and stated that he had had the honor of continuously serving on the Board of Censors since January 12, 1868, and that he now desired to be relieved from further duty on this board. Several members tried to urge the doctor to reconsider his determination. It was intimated that inasmuch as he had served forty (40) years, it would be an exceptional honor to round out a fifty (50) years service. Dr. Hopkins, however, stated that he was fully determined upon his course and would have to decline further service, even if elected. He thanked the society for the expression of good will and for the support given him during his long term of office.

Nominations for censors, in addition to those nominated at the June meeting were then called for by the president, with the result that George L. Brown was nominated. On motion, the secretary was directed to cast the ballot of the society for the following as censors: DeLancey Rochester, John H. Grant, Francis E. Fronczak, Walter D. Greene and George L. Brown. This being done, the president declared them duly elected as censors for the year 1909.

On motion, the secretary was directed to cast the ballot of the society for F. Park Lewis as Chairman of the Committee on Legislation, Ernest Wende as Chairman of the Committee on Public Health, and Thomas H. McKee as Chairman of the Committee on Membership.

The secretary was then directed to read the names of the nominees for Delegate to the Medical Society of the State of New York for the years 1909 and 1910. Ballot was taken and tellers announced the result as follows:

Those elected delegates are Charles A. Wall, Edward Clark, Thomas H. McKee, Arthur G. Bennett, and Eli H. Long. They were declared by the president, as duly elected delegates to the State Society for the years 1909 and 1910.

When election was called for delegates to the 8th District Branch, Dr. Wall stated that under the new rules adopted by the 8th District Branch, there was no longer any necessity to elect delegates, as the rules make every member of the society a delegate.

Election of officers being completed, Dr. Rochester stated that last year there was a misunderstanding as to whether the delegates to the state society were instructed to present a candidate for the position of president, consequently the presidency of the state society was lost to Western New York; that he has assurances that Western New York can have the presidency this year, if it requests it; and if Erie County unites upon the name of Dr. Stockton, he will be supported by Monroe County and be elected; but if not united upon Dr. Stockton, then Monroe County will nominate a candidate of its own.

On motion of Dr. Hopkins, it was *Resolved*, That the Medical Society of the County of Erie instruct its delegates to present the name of Dr. Charles G. Stockton of Buffalo for the presidency of the Medical Society of the State of New York, and to use all honorable means to procure his election. The resolution was unanimously adopted.

Dr. Wall moved that the by-laws relative to election of new members be suspended and that all elected at this meeting become members January 1, 1909. Carried.

Dr. T. H. McKee, Chairman of the Committee on Membership then presented the following application for membership: Norman K. McLeod, 327 Delaware Avenue, Buffalo; Richard R. Gould, 1673 Broadway, Buffalo; William F. Beck, 1379 Clinton Street, Buffalo; Arthur C. Schaefer, 753 Michigan Street, Buffalo; Alfred H. Noehren, 571 E. Utica Street, Buffalo; Vincent B. Beszczynski, 1016 Sycamore Street, Buffalo; H. W. Cowper, 385 Franklin Street, Buffalo; Harry E. Braner, 291 Maple Street, Buffalo; Leonard Reu, 841 Smith Street, Buffalo; Daniel V. McClure, 799 Elk Street, Buffalo; Hiram D. Walker, 735 Main Street, Buffalo.

Each of the foregoing candidates was individually ballotted for, the secretary being instructed to cast the ballot of the society for each candidate, after which the president declared them duly elected to membership.

Dr. McKee then stated that this makes 59 new members elected during the year, and that 48 other persons had been reinstated to membership, making a total of 107 new members for 1908. This creditable showing called for very complimentary remarks, and a hearty vote of thanks was given the membership committee for its good work during the year.

Dr. Hopkins, Chairman of the Board of Censors, then read the annual report, as follows:

### REPORT OF THE BOARD OF CENSORS.

*To the President of The Medical Society of the County of Erie:*

The censors respectfully report that with distinct satisfaction and encouragement, a review of our work for a number of years reveals the fact that an important change has taken place in public opinion regarding our medical laws, and with greater particularity regarding our medical societies charged with the responsibility of initiating proceedings in cases of violations of those laws.

A few years ago, less than a decade, the work of the censors was made exceedingly difficult, and their most efficient work was made impossible by reason of the almost universal belief that medical laws were for the protection of the medical profession, that such laws were invoked by one set of doctors to annoy or to punish a competitor or rival, and that the real motive of the prosecution in most cases was envy and jealousy on the part of the prosecutor for the superior success of the prosecuted,—the persecuted. For many years, public sentiment and opinion as indicated by the public press, by the findings in the rooms of our grand and petit juries, in the offices of our district attorneys and superintendent of police, has run in this attitude adverse, through misunderstanding, to medical laws, to medical societies, and to the work of the censors. It is, therefore, with unfeigned satisfaction and hopefulness that your censors report, that the events of the last year demonstrate that public opinion and sentiment as to medical laws has changed, that misunderstanding has given place to right interpretation, that our people now know that our medical laws are in the interest of and for the protection of our most helpless folk,—the poor, the credulous, the ignorant, the sick. The significance of this improved public sentiment and its bearing upon the future of our society and upon the labors of the censors of the coming years, seems to your board hopeful in the highest degree.

In July last, your censors received a letter from the publisher of the *Buffalo Express* enclosing an advertisement from the paper, also a news clipping, asking our opinion as to the accuracy of the statements of both, advertisement and clipping. This advertisement,—that of the Oxygenator Company, E. L. Moses, General Manager, 522 Brisbane Building, Buffalo, N. Y., is a fair sample of the average work of the medical fakir. The promoters of this device took advantage of the presence in the city of scarlet fever, and that in an unusual degree, to scare the public into the purchase of their expensive instrument claiming for it marvelous powers both for cure and prevention.

The moral level of this particular medical fakir was indicated by the fact that he pretended to quote Dr. Gram, of the Bureau of Vital Statistics, as authority for the statement that there had been in our city in June, the last month, 154 cases of scarlet fever, with 34 deaths, the fact being that there had been 154 cases, with four deaths. The same false statement as to the number of deaths also appeared in the clipping. Of course, it was easy to show the publishers of the *Express,* the official statement of Dr. Wende as to the pertinent facts in the case, to prove the abominable falsity of the oxygenators in both clipping and advertisement, and the latter ceased to appear. The significance of this incident is that the publisher of the *Express* voluntarily sought the help of the censors in order that the paper might be free from the responsibility of aiding the nefarious work. Congratulations to the *Buffalo Express* would seem to be in order.

Many of the more successful fakirs use some form or counterfeit of religion both as an advertisement and as a method or means of treatment. Antonius, the boy wonder, was a conspicuous example of this, and there are many others.

About the middle of May last, there came to town a pair of medical fakirs, of the over-developed religious type. These healers advertised themselves as Pope Messiah and Brother Schlatter. They opened an office and prepared for the business of healing for revenue at 77 Swan Street, their last place of business, where it is said they had a miraculous success, having been at Saint Louis. It should be remarked that the blasphemous conduct of this precious pair of healers, as indicated by their titles was consistently, even extravagantly, supported by their language, manners and clothing,—sacred gestures, sacred vestments,—sacred words were profaned and prostituted to the furthering of their criminal purposes and that systematically and persistently. Here was an admirably devised plan for repeating the years and years of the pecuniarly successful faking of Antonius, the boy wonder, the miracle worker.

The result was quite different. The attention of the censors was invited to the so-called, self-styled Pope Messiah and Brother Schlatter by a reporter from the *Buffalo Evening Times,* with a request for information as to the legal status of these men and their work. The provisions of the law bearing upon the case were cited, the criminal character of the work was pointed out, the nature of the evidence necessary to convict was indicated. The further work of the censors in the case included a call upon District Attorney Abbott and a call upon Superintendent of Police, Regan. The result was that after a stay in town of less than a week, the fakirs were summoned to police headquarters where Brother Schlatter appeared, (the self-styled Pope Messiah having fled the town the previous night), and was ordered to leave town quick.

We get an idea of the pecuniary value to a city like Buffalo of a right judgment as to medical laws, when we contrast the intelligent handling of the fakirs, the self-styled Pope Messiah and Brother Schlatter, with the years and years of immunity enjoyed by the so-called Antonius, the boy wonder, the miracle worker, and the final expense to the county and state incidental to his conviction and imprisonment.

Truly public opinion has grown decidedly from the day of Antonius to the day of Brother Schlatter. Again, sincere congratulations to the *Buffalo Evening Times,* to District Attorney Frank A. Abbott and the Superintendent of Police, Michael Regan, are most emphatically in order.

We have alluded to the exceeding hopefulness of the future of the medical profession as represented in its executive societies, and believe that three features are worthy of your consideration:

(1) The strengthening of our public health law by the enactment of a section as shown in Chapter 344, Laws of 1907, defining in accurate, comprehensive terms what is the practice of medicine, enlarges and makes hopeful the protective work of our society. The medical fakirs and criminals are slow to believe, and find it difficult to accept the sweeping provisions of our present law: However, there is every probability that the courts will sustain these provisions, not only as law, but as law which is both wise and humane.

(2) The government at Washington, on June 30, 1906, approved a law "For preventing the manufacture, sale, or transportation of adulterated or misbranded or poisonous or deleterious foods, drugs, medicines, and liquors." The strong words in this law are the words "misbranded" and "label", the provision regarding the latter being "Descriptive matter upon the label shall be free from any statement, design, or device regarding the article or ingredients or substances contained therein, or quality thereof, or place or origin, which is false or misleading in any particular."

"The use of any false or misleading statement, design, or device, shall not be justified by any statement given as the opinion of an expert person or appearing on any part of the label, nor by any descriptive matter explaining the use of the false or misleading statement, design or device." "That the term 'misbranded' as used herein shall apply to all drugs, the package or label of which shall bear any statement, design, or device, regarding such article, or the ingredients or substances contained therein which shall be false or misleading in any particular." The law further provides that any person who shall ship or deliver for shipment, receive, sell or offer for sale any misbranded drugs or medicines shall be guilty of a misdemeanor and liable to fine and imprisonment.

The significance of the provisions of our national food and drug act comes to us with greater clearness when we recall the intelligent supervision given to what may and to what may not circulate through the mails.

(3) In the medical law of our state in 1907, most important powers are given to the regents for maintaining discipline in our profession. These powers run in the direction of revoking or annuling license, an enlargement of those previously given. Formerly the regents might revoke a license when the holder had been convicted of a felony; now, power is given to revoke or annul for a wide variety of misconduct.

The significant facts in the new law are the widening of the causes for discipline and the more important provision, that these trials shall take place not before our courts but before a court of medical men, our board of medical examiners. We think we are not unduly optimistic when we declare to you our conviction that the future of the medical profession as to the preparedness of the new members, and as to the discipline of all its members, was never more hopeful than at the present time. During the last year two members of our profession,—Messrs. Turver and Ince,—have concluded their terms of imprisonment in two of our penal institutions; have found their licenses annulled, and have approached the censors with the proposition of an application to the regents looking to a resumption of professional work. The question is one of considerable importance to the applicant and to the profession at large.

It is the opinion of the censors that every such case should be decided only after full hearing, with ample time for preparation, and we recommend that the secretary be instructed to request from the regents notice of at least two weeks in case any person living in this county makes application for license under these conditions.

During the year several complaints have been made for violations of the public health laws, which are now under consideration. The society may expect to hear of them in subsequent reports. Our work has required frequent consultations with our counsel, John Lord O'Brian, and his competent opinions have always been given freely and gladly.

We have also, as in many previous years, received valuable advice, upon many occasions from District Attorney Abbott and from Assistant District Attorney Murphy. The obligations of the medical profession to these gentlemen, we have, on previous occasions, tried to express. We have the profound convictions that these obligations can never be over-acknowledged. Our thanks, as in former years, are due to our efficient Superintendent Chief of Police Michael Regan, his aid and coöperation have been invaluable.

Respectfully submitted,

(Signed) HENRY REED HOPKINS,
JOHN HUDSON GRANT,
FRANCIS E. FRONCZAK,
DELANCEY ROCHESTER,

December 21, 1905.

On motion of Dr. Rochester, the report of the Board of Censors was received and recommendations contained therein were adopted.

Dr. Lytle submitted his annual report as treasurer of the society, as follows:

When elected to fill this important office, I was led to believe it to be a sinecure and a lazy man's job, but upon consulting the bylaws of both the state and county societies, and studying the membership ledger turned over to me by my predecessor, I found that the lazy man's job was a myth, and that if I expected to come anywhere near to doing my duty as Treasurer of the Medical Society of the County of Erie, I would have to get busy.

The membership ledger handed over to me was of the old cumbersome type requiring much time to keep, so, with the permission of the council, a new and shorter system was adopted. The accounts in the old ledger were unbalanced, often simply scratched off with meager entries as to authority for so doing. and many were in dispute, as I discovered later, after protracted correspondence and verbal interviews. State assessments became part of membership obligations in 1906, and the ledger I received showed that 116 members had never paid any state assessments and were owing more or less in back dues to the society, while 24 other members had paid but one state assessment and owed the society more or less dues. Of these 140 members, 96 were owing county dues prior to 1906, anywhere from one to fifteen years.

According to the bylaws, these 140 members were no longer members of the society, but it was assumed that the disturbed conditions brought about by amalgamation of the former association with the society and the union with the state society, all of which, though desirable, were delayed for several years, were the cause of much of the back-sliding on the part of the members. It was thought desirable to make a determined effort to have it out, if possible, with each delinquent member, so a vigorous and protracted campaign was begun, during which, 1,600 pieces of mail were sent out, and, in addition, innumerable telephone conversations and personal interviews were carried on. The results, I think, have been satisfactory, in that the present membership holds open accounts with only 311 members who owe only for 1908, all others having been disposed of as shown in the following table; in that many who felt themselves unjustly dealt with by the society were treated squarely and made loyal; in that many cases of rank unfairness were adjusted; and in that all were awakened to the advantages of and the necessities for active membership in the society.

December 21, 1908.

*Mr. President and Fellow Members of the Medical Society of the County of Erie:*

Upon taking office in January, 1908, and during the year just ending, the treasurer has charged himself with the following assets of the society:

TREASURER, DR.

| | | |
|---|---|---|
| Cash received from preceding Treasurer, | | $ 210.06 |
| Accounts received from precediug Treasurer, State | $1.047.00 | |
| Accounts received from preceding Treasurer, County,. | 1,132.75 | |
| | | 2,179.75 |
| Dues and assessments for 1908, due Jan., 1908, State | 813.00 | |
| Dues and assessments for 1908, due Jan,, 1908, County, | 541.00 | |
| | | 1,354.00 |
| Dues and assessments from new members, State | 144.00 | |
| Dues and assessments from new members, County | 96.00 | 240.00 |
| Treasurer owing honorary, etc | | 12.00 |
| Total chargeable to December 31, 1908 | | $3,995.81 |

During the same period of time, the treasurer has credited himself as follows:

TREASURER, CR.

| | | |
|---|---|---|
| Orders No. 1 to No. 104, inclusive, distributed as follows: | | |
| State treasurer, direct | $930.00 | |
| State treasurer, indirect (rebates) | 192.00 | |
| | | $1,122.00 |
| Secretary | | 184.02 |
| Treasurer | | 110.03 |
| Censors | | 104.00 |
| Membership | | 41.36 |
| Municipal Hospital committee | | 7.46 |
| Sundry items | | 59.00 |
| Total Cash | | $1,627.87 |

By cancelled dues and assessments:

| | | | |
|---|---|---|---|
| Death preceding 1908, State | $72.00 | | |
| Death preceding 1908, County | 80.00 | | |
| | ——— | $ 152.00 | |
| Death during 1908, State | 3.00 | | |
| Death during 1908, County | 2.00 | | |
| | ——— | 5.00 | $ 157,00 |
| Resignation, State | | 153.00 | |
| Resignation, County | | 130.00 | |
| | | ——— | 283.00 |
| Reinstatement, State | | 60.00 | |
| Reinstatement, County | | 47.50 | |
| | | ——— | 107.50 |
| Suspension, State | | 570.00 | |
| Suspension, County | | 633.75 | |
| | | ——— | 1,203.75 |
| Dropped for non-payment of dues, 1908 | | | 25.00 |
| Total cancellation | | | $1,776.25 |
| Total cash | | | 1,627.87 |
| Total to be credited to December 31, 1908 | | | $3,404.12 |
| Leaving balance on hand (December 21, 1908) | | | 591.69 |
| | | | $3,995.81 |

The Cash Accounts show the following Receipts and Disbursements:

December 21, 1908,

RECEIPTS.

| | | |
|---|---|---|
| From preceding treasurer | | $ 210.06 |
| From State assessments, back | $192.00 | |
| From State assessments, 1908 | 939.00 | |
| | ——— | 1,131.00 |
| From County dues, back | 253.50 | |
| From County dues, 1908 | 625.00 | |
| | ——— | 878.50 |
| Total | | $ 2,219.56 |

DISBURSEMENTS

| | | |
|---|---|---|
| Orders No. 1 to No. 104 inclusive, and as per sheet showing treasurer's credits | $1,627.87 | |
| Balance cash deposited to credit of Medical Society of the County of Erie | 591.69 | |
| | ——— | $2,219.56 |

MEMBERSHIP

| | | | |
|---|---|---|---|
| The old ledger contained a membership record of | | 480 | |
| For various reasons, there were scratched off | | 119 | |
| Leaving open accounts | | | 361 |
| Those in good standing January 1, 1908 | | 221 | |
| Those in arrears January 1. 1901 | | 140 | |
| | | —— | 361 |
| Reinstated | | 48 | |
| Suspended | 72 | | |
| Subsequently reinstated | 3 | | |
| | — | 69 | |
| Resigned | | 16 | |
| Dead—from 1 to 6 years | | 7 | |
| | | —— | 140 |

MEMBERSHIP LEDGER, 1908

| | | |
|---|---|---|
| In good standing, from old ledger | 221 | |
| Reinstated | 48 | |
| New members, 1908 | 48 | |
| | | 317 |
| Died during 1908 | 4 | |
| Removed during 1908 | 1 | |
| In arrears, to be dropped December 31, 1908 | 5 | |
| In good standing December 31, 1908 | 307 | |
| | | 317 |

Respectfully submitted December 21, 1908.

ALBERT T. LYTLE,
Treasurer.

The Treasurer's report was received, and the thanks of the society was tendered to Dr. Lytle for the very painstaking efforts he had made in behalf of the society during the year. On motion, the report was referred to an Auditing Committee composed of Drs. Ullman, Bennett and Bonnar, appointed by the president.

Dr. Rochester made a verbal report regarding the subject which had been referred to him at the last meeting, relative to the antispitting ordinance. He stated that, because of the formation of an antituberculosis society which would take up such matters, his committee had dropped the subject. On motion, report was received and committee discharged.

Dr. Grosvenor, Chairman of the Committee on Neurology, presented memorials on the deaths of Drs. Clara E. Bowen, William H. Chase, Edward H. Tweedy, Grosvenor R. Trowbridge, James M. O'Neil, Edward H. Ballou, and Joseph Fowler.

The memorials were unanimously adopted.

Dr. Bonnar, Chairman of the Committee on Municipal Hospital for Infectious Diseases, reported as follows:

December 21, 1908.

*To the Medical Society of the County of Erie:* The committee of fifteen, of which I have the honor of being chairman, named by your president, following the June meeting of this society, for the purpose of bringing to the attention of the Mayor and Council of Buffalo, the urgent necessity of securing a Municipal Hospital for the care of contagious diseases, are now pleased to be able to report to this society that our efforts have been crowned with success.

Following the appointment of your committee on this hospital question, due attention and study of its duties and responsibilities were given the matter by the members generally, who heartily responded to the call of the chair to attend its various meetings called during the summer months. At the first meeting of the committee in the latter part of July, a sub-committee of three was appointed, which consisted of Dr. DeLancey Rochester, Chairman, and Drs. Hayd and Hanley, who were asked to study the problem as to cost, location and such facilities in other cities at home and abroad. This, they performer with a singleness of purpose, and with such efficiency as met with the hearty approval of the committee of the whole, and I wish to formally express our just appreciation for their valuable data.

In the month of October,—following the harvest vacation of the civic boards,—due notice of our desires and that of many allied civic bodies, was filed with the City Clerk and, in the routine of such business, was referred to the Committee on Sanitary Affairs of the Board of Aldermen. A public hearing was given in due process by this committee. At this hearing many of the members of your committee appeared, also a goodly number of the members of this society lent their presence and moral influence to the furtherance of this subject. Right here, I take occasion to state to this society, what is already well known, that in all the deliberations and practical workings of the committee of this society, we were ably supported by the committee of the Academy of Medicine, consisting of five members, of which Dr. Julius Ullman was chairman.

At the hearings before the sanitary committee of the Aldermen, addresses upon the vital necessities of this proposed hospital for contagious diseases, were made by the president of this body, Edward Clark, myself as chairman of committee, Julius Ullman, DeLancey Rochester, R. R. Ross of the General Hospital, Franklin C. Gram, Charles R. Borzilleri, Henry R. Hopkins, P. W. VanPeyma, and Francis E. Fronczak, Assistant Health Commissioner, who, in the absence of the Health Commissioner, Ernest Wende, filed a letter of the commissioner with the committee on sanitary affairs, giving his views upon the imperative needs for such an hospital to save the lives and conserve the interests of homes in our city.

Supplementing these more purely medical arguments, there were not lacking the civic and material interests of our medical men voiced by those whom I have above named, but also and with much added zeal, from purely civic and humanitarian rights, many representatives of civic associations, as the Manufacturers' Club, United Trades and Labor, East Buffalo Business Men's Association, Cigar Makers' Union, School Principals' Association, Federation of Women's Clubs, Mothers' Club, Nurses' Association, The Medical Clinic (composed of female physicians), Teachers' Association, and other patriotic bodies of our citizens, male and female, whose parental and human sense arose as one voice to demand the earliest possible way of escape from a condition well nigh intolerable in a civilised community. The attendance of our citizens on that and subsequent hearings before the Aldermanic Board and the Council of our city was large and representative of the people as a whole, and demonstrated the patent truth that "where there's a will, there's a way," and what the people really need they can obtain if they are willing to work for it. Another inference is apparent,—namely, it is always good economy to procure a necessity where means are available.

Before closing this report, I wish to express the hearty thanks of this committee to the many civic bodies which so unselfishly and ably coöperated with your committee in bringing this matter to a conclusion, finally reached by the Common Council and Mayor of this city, to order the draft of a bill by the Corporation Counsel to be presented to the state legislature, to authorise the city of Buffalo to bond itself in the sum of Two Hundred and Fifty Thousand Dollars ($250,000), to be used in the matter of procuring for our city a Municipal Hospital for the care of contagious diseases.

Feeling gratified at our good fortune and trusting in the full fruition of this most laudable and crying necessity for the efficient care of those afflicted by diseases, not due to any fault of the sufferers, but due, in great part, to just such lack of sanitary facilities for segregating the sick from the healthy, we feel that such outlay, if only to faithfully isolate before the time of contagion was reached, and thus needed by but few of the present numbers, would be such a monument to civic virtue as will redound to the lasting credit of the society and all other bodies of citizens who made this great public necessity their private and public duty to secure.

Respectfully submitted,
Signed by the Committee.

The report was received and filed.

Dr. Ullman said that of the many societies and unions who had helped in procuring the desired results in the matter of contagious disease hospital, Mr. John Hadida, president of the cigar Makers' Union was one of the most active. As Mr. Hadida was present at this meeting, Dr. Ullman desired that the privilege of the floor be extended to him.

The president having granted such privilege, Mr. Hadida made a few brief remarks relative to the subject in which his union was deeply interested. At the conclusion of his remarks, he was heartily applauded.

Dr. Grant moved the following resolution which was unanimously adopted.

*Resolved,* By the Medical Society of the County of Erie, that, in the interest of the health and morals of the community and the prevention of quackery and deception, the coöperation of the newspapers is requested, in not printing, as advertisements, cure-alls and the like fake medicines. That if in doubt as to the propriety of such advertisements, their representatives consult, before publishing them, the chairman of the board of censors, in order that, if wrong, misleading and deceptive, such advertisements be suppressed.

Dr. Bonnar said that since Dr. Hopkins had worked so faithfully for forty (40) years as a censor, it was due to him, in addition to a hearty vote of thanks which he hereby moved, that the society give some tangible expression of the appreciation of the work which the doctor had done for the benefit of humanity. He, therefore, offered the following resolution:

*Resolved,* That this society extends a vote of thanks to Dr. Henry R. Hopkins for the faithful services which he has rendered this society and the community as a censor during the forty years just passed; and that the secretary be instructed to convey this expression of appreciation to Dr. Hopkins.

Dr. Wall moved an amendment that the council be directed to procure a suitable gift, in their discretion, in recognition of the services he had rendered the community and that the amount to be used for such purpose be left to the judgment of the council. The amendment was accepted by Dr. Bonnar, and the resolution, as amended, was then adopted.

Dr. Wall presented the name of Dr. Henry R. Hopkins for honorary membership. Under the rules, this must be acted upon by the Council and laid on the table for a future meeting.

On motion of Dr. Briggs, an honorarium of $100 each was granted to the secretary and treasurer for services rendered.

On motion of Dr. Ullman, the secretary was directed to notify all members of assembly and all senators from this district that they be requested to use all honorable means to secure the passage of the bill for Municipal Hospital for Contagious Diseases.

Adjournment was then taken.

The evening session, which was devoted to scientific purposes only, was called to order by President Clark at 8.30 o'clock, and the following program was carried out:

Ten minute talks as follows:

Roswell Park, The new fulguration treatment of cancer.
Earl P. Lothrop, Papilloma of the bladder.
W. L. Phillips, Treatment of congenital argamblyopia.
E. T. Meyer, Surgical treatment of acute pancreatitis.
L. S. Beals, Lumbar puncture.
N. K. McLeod, Immunity.

At the conclusion of the program Dr. Clark briefly thanked the society for the courtesies extended to him during the year, and he then introduced Dr. Charles A. Wall as his successor.

Dr. Wall was greeted with applause on assuming the chair.

There being no further business, the meeting adjourned.

